# Supplementary material for: Impact of body mass index on pathological response after neoadjuvant chemotherapy: results from the I-SPY 2 trial
Source: Breast Cancer Res Treat. 2024 Jan 12;204(3):589–97. doi: 10.1007/s10549-023-07214-5 (PMC10959799; doi:10.1007/s10549-023-07214-5)
Supplement: Supplementary file 1 — Supplementary file1 (DOCX 18 kb) [file 10549_2023_7214_MOESM1_ESM.docx]

**Supplemental Tables**

**Supplemental Table 5:** Association of BMI with overall survival (hazard ratios, HR)

| **BMI** | **Adjusted HR*** | **95% CI** | **P** |
| --- | --- | --- | --- |
| Normal / Underweight | 1 (Ref.) |  |  |
| Overweight | 1.13 | 0.69-1.87 | 0.63 |
| Obese | 0.82 | 0.46-1.44 | 0.48 |

* Adjusted for age, hormonal cancer subtype, stage, and menopausal status

**Supplemental Table 2:** Association of BMI with Event-free Survival

| **BMI** | **Adjusted HR*** | **95% CI** | **P** |
| --- | --- | --- | --- |
| Normal / Underweight | 1 (Ref.) |  |  |
| Overweight | 0.99 | 0.67-1.49 | 0.98 |
| Obese | 0.88 | 0.57-1.35 | 0.56 |

* Adjusted for age, hormonal cancer subtype, stage, and menopausal status
